# The Nature, Pathology and Treatment of Dipsomania

**Published:** 1881-10-20

**Authors:** Edward C. Mann

**Affiliations:** New York, Physician to Sunnyside, a Private Hospital for the Treatment of Dipsomania, the Opium Habit and Nervous and Mental Diseases


### Southern Medical Record:

EDITORS:

T. S. Powell, M.D. W. T. Goldsmith, M.D. R. C. Word, M.D.

*R. C. WORD, M.D., Managing Editor.*

☞All Communications and Letters on Business connected with the Record must be addressed to the Managing Editor.

Vol. XI. ATLANTA, GA., OCTOBER 20, 1881. No. 10.

### ORIGINAL AND SELECTED ARTICLES.

### THE NATURE, PATHOLOGY AND TREATMENT OF DIPSOMANIA.

BY EDWARD C. MANN, M.D., OF NEW YORK,

Physicirn to Sunnyside, a Private Hospital for the Treatment of Dipsomania, the Opium Habit and Nervous and Mental Diseases.

Dipsomania, or periodical inebriety as many term it, is to be classed under the head of the psychical degeneration states affecting the brain, and belongs in the subdivision of periodical insanity. It is characterized by an incontrollable and intermittent impulse to take alcoholic stimulants or another agent, as opium or chloral, which causes intoxication. We must distinguish between this and the physiological state, in which the individual merely chooses to indulge in liquor to excess. The great question of importance is to distinguish the two states or conditions, when the result—inebriety—is the same. We must observe whether there are symptoms in our patient which can be referred to primary disease of the nervous system. We must examine for hereditary influences, which when present lead us, of course, to suspect disease. Early development of the appetite for stimulants points in the same direction ; but the great diagnostic point attending the *disease* is the *irresistible* impulse by which the patient is impelled to gratify his morbid propensity, being, during the paroxysm, blind to all the higher emotions, and pursuing a course against which reason and conscience alike rebel. It is frequently seen that these paroxysms are preceded by considerable disturbance of the nervous system. The patient perspires and is sleepless, uneasy and prostrated, and so craves some stimulant. Between the paroxysms he is different from a common drunkard, in oftentimes disliking exceedingly all stimulants, and is then a useful member of society.

Dipsomania has been described under three forms: acute, periodic, and chronic. The acute form is the rarest, occurring only after exhausting diseases or excessive venereal indulgence. The periodic form is much more frequent, and is observed in persons who have suffered injury to the head or spine, females during pregnancy and at the catamenial period, and also in men whose brains are overworked. This form is frequently hereditary, and consequently proportionately difficult of cure. These patients may abstain for weeks and months from all stimulants, and may, during this interval, positively dislike them. At last, however, the patient becomes uneasy, listless and depressed; is not inclined to apply his mind, and finally begins to drink and continues until intoxicated. The patient continues this course for ten days or perhaps a forthnight, and then bitterly regrets his fall. This often runs on, if not checked, into mania and lapses into dementia.

The chronic form is the most incurable form of the disease, as the patients are incessantly under the irresistible desire for alcoholic stimulants. I think the latter class of cases require constant seclusion in an asylum, if they wish to be free from intoxication, as a discharge or leave of absence is always followed by a repetition of the same acts. In a majority of cases of this nature we find hallucinations of sight and hearing, which oftentimes produce very painful moral impressions and at times even great terror in the patient. Cases of delirium tremens are excluded in these remarks. These patients manifest confusion of thought, perversion of feelings, suicidal tendencies, tremors of the facial muscles and tongue, anæsthesia of the extremities at times, and very olten paralytic symptoms going on to general paralysis.

The subject of hereditary metamorphosis of the diseases of the nervous system is of great importance in this connection. As a result of intemperance in the progenitors, we find transmitted to the offspring, allied but different forms of neuroses. It may be dipsomania, epilepsy, chorea or actual insanity, or a proclivity to crime. It is, at all events, an aptitude for some form or other of nervous disorder, the particular form being often determined by causes subsequent to birth.

The law of hereditary transmission applies equally to the victims of dipsomania as well as to the other insane classes, and is to be studied, I think, in three divisions according as it is manifested.

1. In predisposition or simple aptitude, the result of a defective organization, and a weakened or diseased nervous system, as a result of which the possessor is predisposed, or has a tendency to seek for the relief obtained by alcoholic stimulants when laboring under physical or mental depression.

2. The latent state or germ of the disease, and

3. In the actually developed disease.

The first of these states, the predisposition or aptitude, being hereditary to a strong degree, is universally acknowledged to be the most difficult to eradicate, and requires the wisest sanitary conditions adapted to both mind and body. Most people doubt the existence of the seeond or latent state or germ of the disease, ignoring the law of progressive development, and such persons find it difficult to believe that dipsomania coming on in maturity, as a result of ill-health, mental shock, etc., may have originated in intemperance in the parent or grand parent. Yet this is a fact. One very important organic law which should be universally understood in this connection is that morbid impulses and characteristics and traits may disappear in the second generation and break out with renewed intensity in the third, although a tendency or predisposition may be transmitted to the offspring, and under good hygienic and other favorable circumstances die out and fail to be transmitted any further.

I have remarked in my experience with the insane, whether the exciting cause be intemperance or something else, that the cases most unlikely to recover are those where the insane temperament or diathesis is clearly manifested and where the predisposition to disease is inherited. Such patients, though they may have lucid intervals, rarely if ever entirely recover. I think the insane impulses to drink, which overcomes all the efforts of the individual who inherits a tendency in this direction, present the same indications for treatment as do the suicidal and homicidal impulses, namely, seclusion from society, and the necessary restraint in an institution.

I do not agree with that class of persons who hold that under all circumstances the dipsomaniac is to be treated as an invalid, with the utmost kindness and forbearance, and then, with the strangest perversity, turn round and tell you that inebriety is no excuse for criminal actions, and fine and imprison the unhappy man who has been driven into the debauch by an irresistible craving for drink, when properly he should be regarded as insane, and should be sent to an inebriate asylum for treatment and cure.

Our laws, at present, fail lamentably in preventing intemperance, and this is due, in a great measure, to the false view in which this disease is held by the judiciary. The different forms of dipsomania correspond in their manifestations and oftentimes in their causes, to other cases of mental disease, and cannot properly, I think, be separated from them as regards the fact of the disease. In my views I am upheld by the authority of the eminent Professor Krofft-Ebing, of Germany, who, in his "Handbook of Psychiatry" recently published, which is one of the best works extant on insanity, classes dipsomania with the periodical insanities. Dipsomania often appears as a result of the same causes that operate in the production of other types of mental disease, such as ill-health, severe mental shock, blows on the head and spine and sun-stroke. I consider it, in common with constitutional affections, insanity; moral insanity—the monomanies—epileptic insanity—hysterical insanity and hypochondriacal insanity, one of these physical degenerative states affecting the brain, injured by hereditary or acquired vices of conformation or nutrition.

The depraved alcoholic craving in dipsomania is an irresistible impulse which the mind seems powerless to control; an insane impulse, just as surely as a homicidal or a suicidal impulse is an insane impulse, and one cerebral pathology is equally applicable to this as to other forms of insanity. The terrible insane craving for alcoholic stimulants is often the result of a lowered vitality or abnormal organic development of the nervous system that has descended from generation to generation, gaining in intensity until it manifests itself by the complete loss of self-control and action, inebriety in children and grandchildren, after they once taste intoxicating liquors and indulge in them.

The blunted moral perception which so many inebriates exhibit, and which renders them peculiarly liable to a relapse after they leave an asylum, is to be regarded in the same light, I think, as the perverted moral sense in moral insanity. In every institution for the insane, we find inmates who exhibit no obvious intellectual aberration or impairment, the moral faculties being deranged, while the intellectual faculties remain apparently in their normal condition. The manifestations of moral insanity may be a simple perversion of some sentiment or propensity under certain exciting causes; and I think this exactly comprehends cases of dipsomania with loss of self-control and perversion of the moral sense. The person, of course, is aware that the act is wrong in both instances, but the control which the intellect exercises over the moral sense is overdone by the superior force derived from the desire. I have been told many times, by both insane patients and dipsomaniacs, that the feeling on the one hand to commit some insane deed, and on the other to give way to alcoholic craving or appetite, was contemplated in both instances with horror and disgust, and at first successfully resisted, until at last, having steadily increased in strength, it bore down all opposition. What can be a more powerful argument in favor of the disease theory of dipsomania ?

We find in dipsomania the general symptoms of exhausted nervous power, viz: general debility of the body, inability to walk, even short distances, without fatigue; general feeling of languor; unwillingness to make any active exertion; great tendency to sweat, more especially at night, but also induced during the day by the slightest exertion, and often an unsteady gait. I have found these patients exceedingly prone to neuralgia. The explanation of this is probably due to the fact that there exists in such cases a worn, irritable, hyper-sensitive condition of the sensory nerve-cells of the central senory tract, which is the sole seat of true nervous sensibility. The central nervous system is affected beyond all doubt by excessive drinking, and the degeneration thus produced I regard as a powerful pre-disposer of neuralgia of the inveterate type. Aside from the direct influence impressed on the nerve-centers, I think that this irritable and hyper-sensitive condition of the central sensory tract is often induced by visceral irritative disease of the stomach, kidneys or liver, so common in inebriates, which almost necessarily affects the sensory nerves which ramify in these organs, and from these diseased nerves a more or less steady stream of irritative and wearing nervous impressions is transmitted, practically without cessation to certain parts of the sensory tract, to which the sensory nerves from any given part may go, and, as a result, sooner or later the central sensory-cells are brought into that degree of nutritional disturbance which is the fundamental factor in neuralgia. The real seat of these severe neuralgias, from which so many dipsomaniacs suffer, is rarely, if ever, in the peripheral nerves of the affected region, but in the central nerve apparatus. The heart's action is weak, often irregular, accompanied by palpitation and not unfrequently with symptoms of indigestion.

A change has also come over the man's mind, so that the very *morale* of the mind is changed. At one moment he may be very joyous and excitable, and then he will become greatly depressed. He will be very friendly and anon very hostile. He will be so obstinate that nothing can overcome his determination, and at other times you may lead him like a child. The heretofore ever ready and resolute man manifests marked indecision of character, and in other cases there may be an utter inability to fix the mind on any one subject, or to follow up a train of thought consecutively. Not alone is there a loss of tone in character and blunting of moral perception, but intellectual discrimination is much impaired, and impairment of all the mental faculties is almost inevitable. The ideas are more spontaneous, less under the power of control, and any exertion requiring continuous mental effort soon becomes impossible. There can be no doubt in the gravest cases, that alteration of the brain is taking place *pari passu* with these alterations of character. It may be atrophy, or the circulation through the brain may be checked or impeded by ossification or softening of the cerebral arteries or some disease of the heart itself, or the nervine or brain substance, may be undergoing a change, particularly on its peripheral surface as well as on the surface of its ventricles or cavities. The convolutions of the brain become paler and the furrows shallower. The weight of the whole cerebrum and cerebellum is lighter and less complex. Softening of the brain of a very delicate nature may be taking place; or, what is very likely, and is often passed by unnoticed, becomes discernable only to a well practiced eye, which may not be present at the right moment for observing its attack, is a very slight fit of apoplexy and paralysis, so slight indeed, that it occurs and passes away unnoticed and unperceived, and is recognized only in its after consequences and permanent effects. From the date of such an occurrence, though loss of life does not ensue from it immediately, yet in its ultimate effects it is sooner or later fatal. The patient is an altered man and never recovers himself. So delicate is the tracery of the nervous structure that the damage of a single fibre or set of fibres, destroys the unity of the whole.

There are generally three things present that lead to these attacks of cerebral hemorrhage, and as these attacks play a very important part in the production of premature mental decay in inebriates, it is desirable to thoroughly understand them and estimate their importance, and I will try to briefly explain them. The three factors I allude to are, hypertrophy of the left ventricle of the heart; chronic diseases of the kidneys; and thirdly, degenerated cerebral arteries. The hypertrophy of the heart is a simple hypertrophy of the left ventricle, the walls of the ventricle being thickened without any dilatation, although in exceptional instances dilatation may ensue. The blood, in inebriety, is more or less noxious to the tissues, since it contains an alcoholic foreigner, and its passage into the capillaries is undoubtedly resisted by contraction of the small arteries, the vessels most rich in muscular tissue. The muscular coats of these vessels therefore are hypertrophied in antagonism to the heart. Since the small arteries are hypertrophied throughout the body, the obstructions, though each is slight, are, in their sum-total, so large that in order that the circulation may be carried on efficiently, hypertrophy of the heart must ensue. There may be, doubtless, degenerative changes in the small arteries, so that there may be increased bulk with altered structure. It should not be assumed, I think, as it often is, that all the processes in the arteries eading to cerebral hemorr hage and apoplexy are of a degenerative origin, as there can be no doubt that the presence of alcohol sets up a condition of sub-inflammatory irritation which plays a very important part in the production of cerebral hemorrhage. The sub-inflammatory irritation causes the arteries to lose much of their elasticity and to become permanently wider, larger and more tortuous. This absence of elasticity of the larger arteries becomes, by the withdrawal of the aid to the circulation in equalizing the flow of the blood, an important factor in leading to rupture of the small arteries.

When the brain wastes slowly, as it often does, the dilatation of the vessels and the increase in the quantity of the cerebro-spinal fluid favor rupture very decidedly. There can be no doubt that the occurrence of cerebral hemorrhage in inebriates, resulting from abnormal strains, would be much more frequent were it not for the provisions which nature has made for the protection of the brain from suddenly increased afflux. The turns of the carotid and vertebral arteries, the free anastomosis of the circle of Willis, and the small size of the arteries beyond that circle, before they enter the brain substance, all tend to protect the brain. The perivascular canals also exercise a protective influence over the vessels they surround, and in the corpus straitum, where cerebral hemorrhage is especially liable to occur, as its vessels are not capillary in size and proceed from the middle cerebral artery, which is almost the continuation of the internal carotid. We find the perivascular sheaths of very large size. When I say, then, that I consider one of the principal causes, if not the principal cause, of premature mental decay occurring in inebriates to be the occurrence of cerebral hemorrhage resulting from degeneration caused by the poisonous effects of alcohol upon the tissues, I do not think I overstate the actual facts. We generally have associated in such cases, hypertrophy of the left ventricle of the heart, as I have previously remarked, chronic disease of the kidneys and degenerated arteries. The strong left ventricle and inelastic arteries combine to prevent the wave of blood sent to the arteries from being properly equalized, and consequently the smaller arteries of the brain, which are normally thinner than the arteries of other parts, and which are degenerated, receive the impulse from the heart's jerks, and being thus diseased and fragile—perhaps dilated and aneurismal, give way.

[*Concluded in next number.*]

